# Investigating the association between severity of COVID-19 infection during pregnancy and neonatal outcomes

**DOI:** 10.1038/s41598-022-07093-8

**Published:** 2022-02-22

**Authors:** Anita Dileep, Sham ZainAlAbdin, Salah AbuRuz

**Affiliations:** 1 Department of Primary Healthcare, Women Health Unit, Dubai, United Arab Emirates; 2 Department of Pharmacology and Therapeutics, College of Medicine and Health Sciences, The United Arab Emirates University, Al Ain, United Arab Emirates; 3 Department of Clinical Pharmacy, Faculty of Pharmacy, The University of Jordan, Amman, Jordan

**Keywords:** Diseases, Infectious diseases, Viral infection

## Abstract

Pregnant women with COVID-19 require special attention and care, since the infection does not only affect the mother, but also her neonate and adversely affects pregnancy outcomes. The main goal of this retrospective cohort study is to investigate association between the maternal COVID-19 severity and risk of developing adverse neonatal outcomes. Patients were stratified into asymptomatic/mild and moderate to severe COVID-19. The following neonatal outcomes were assessed: gestational age at the time of delivery, birth weight, neonatal infection, neonatal intensive care unit (NICU) admission. The average age of patients was 28.5 ± 1.4 years old and majority were multigravida (74.0%, n = 148). Of total 200 pregnant women with COVID-19, 26.5% (n = 53) had moderate/severe disease and presented with higher incidence of preterm delivery and low birth weight (88.7%, n = 47; p < 0.001). In addition, more than half of the newborns delivered by mothers with severe disease were infected by SARS-COV-2 (58.5%, n = 31) and majority were admitted to the NICU (95.0%, n = 52). Based on the multivariate logistic regression analysis, pregnant women with moderate to severe COVID-19 were at much higher risk of preterm delivery, lower birth weight, neonatal infection, as well as neonatal ICU admission (p < 0.001). In addition, multigravida women were at higher risk for preterm delivery and lower birth weight (p = 0.017 and p = 0.02; respectively). Appropriate protective measures and early detection of suspected COVID-19 should be addressed for more favorable obstetric outcomes.

## Introduction

The emerged severe acute respiratory syndrome coronavirus 2 (SARS-CoV-2) has caused unprecedented crisis over the world since the beginning of 2020. Pregnant women are among one of very sensitive population that require particular attention during emergencies and infectious diseases. Several studies revealed that infected pregnant women acquire more severe form of COVID-19, by which nearly quarter of them develop pneumonia and have an increasing risk of mortality up to 35.0%^1,2^. Additionally, the Centers of Disease Control and Prevention (CDC) reported that pregnant women with SARS CoV-2 infection disease-19 (COVID-19) were more likely to be hospitalized compared to non-pregnant at an equivalent age (31.5% versus 5.8%)^3^. Severe illness appears to be more common in later pregnancy as reported by the UKOSS study, where most pregnant women were hospitalized in the third trimester or peripartum^4^. According to the literature, the severity of maternal disease and obstetric outcomes can be attributed to the immunologic and physiologic alterations during pregnancy including low respiratory capacity, vascular and hemodynamic changes, increased oxygen demand, risk of aspiration, decreased maternal tolerance to hypoxia, anatomical changes with increased transthoracic diameter and reduced diaphragm height, as well as preexisting health conditions as preeclampsia or diabetes that contribute to a higher risk of developing more than 190 maternal complications^5,6^. Furthermore, there is a potential risk of vertical transmission to fetus or placental infection that may end up with a range of cascade including premature delivery, cesarean, admission to the neonatal intensive care unit (NICU), growth restriction, early onset neonatal sepsis, fetal distress, or pre-term birth abortion^7–9^.

Regarding the clinical presentation of pregnant women with COVID-19, a review of collective studies indicated that the laboratory and radiographic investigations are similar as non-pregnant ladies, however, the most common abnormalities among infected pregnant women were decreased lymphocytes, elevated C-Reactive Protein (CRP) in blood, and pregnancy-related complications including preterm delivery and cesarean section. On the other hand the most reported clinical manifestations were fever, myalgia and cough^8,10,11^.

There is considerable data available on COVID-19 infection among pregnant women in the literature, but only few studies have investigated the association between severity of maternal disease with perinatal outcomes. Currently, a health system for monitoring progression of COVID-19 diseases has been created in many hospitals; therefore, it is essential to collect and record information about COVID-19 disease among pregnant women and its’ influence on maternal and neonatal health outcomes. The goal of this study is to investigate the association between severity of maternal COVID-19 among pregnant women and the risk of adverse neonatal outcomes. Another goal is to examine the impact of demographic factors on neonatal outcomes. We hypothesized that severity of maternal COVID-19 infection has significant impact on neonatal outcomes including preterm birth, neonatal birth weight, neonatal infection, and neonatal intensive care unit (NICU) admission.

## Methodology

### Study setting

This multicenter study was conducted on pregnant women with confirmed COVID-19 at the women health clinics of Dubai hospital, Latifa hospital, and a Primary health Care clinic located in Dubai. The study was approved by the Dubai Scientific Research Ethics Committee (DSREC) and was carried out during a period ranging from January to December 2020. All experiments were performed in accordance with relevant guidelines and regulations. The informed consent was waived by DSREC since the study is retrospective, and patients’ identity was anonymized.

### Participants’ selection and data collection

The study inclusion criteria were (1) Pregnant women at or beyond 24 weeks (2nd and 3rd trimesters) of gestation and (2) With documented diagnosis of COVID-19 Infection. The diagnosis of SARS-Cov2 was established as per the World Health Organization (WHO), using nasopharyngeal real-time polymerase chain reaction (RT PCR). Clinical presentation at the time of positive COVID-19 was observed along with any progress in disease severity throughout pregnancy. Patients’ clinical data was extracted from medical records, which were reviewed electronically till the time of delivery. The following were the exclusion criteria: Pregnancy at less than 24 weeks of gestational age, older mothers above age of 35 years, grand multipara, and patients with any medical comorbidities including morbid obesity (BMI ≥ 40 kg/m^2^). The exclusion criteria were considered since these categories of pregnant women are already at high risk of neonatal outcomes. Therefore, excluding them from the study would minimize the risk from confounders.

Patients were categorized as asymptomatic/mild and moderate/severe according to National Institute of Health (NIH) classification and described below in details^12^.

### Study design

This is a multicenter retrospective cohort study. The main risk factor investigated was severity of illness which was categorized into two the following categories as per the NIH classification.

1. Moderate/Severe group was defined as follows:
  Moderate illness, which shows evidence of lower respiratory disease upon clinical or chest imaging with oxygen saturation (SpO_2_) > 94.0% at room temperature. Severe illness that is associated with low SpO_2_ (< 94.0%), or ratio of arterial partial pressure of oxygen to fraction of inspired oxygen (PaO_2_/FiO_2_) of < 300 mmHg, respiratory frequency of > 30 breaths/min or lung infiltration of > 50.0%.
2. Asymptomatic/Mild was defined as follows:
  Asymptomatic pregnancies with confirmed positive result of COVID-19 using virologic tests and have no symptoms consistent with COVID-19 manifestation.
  Mild cases who have definite COVID-19 symptoms (e.g. cough, fever, sore throat, malaise, headache, muscular pain, nausea, vomiting, diarrhea, loss of taste or smell) without dyspnea or abnormal chest imagining.

The two groups were compared with regards to the below neonatal outcomes. Logistic regression was also conducted to investigate the impact of other variables on neonatal outcomes and to investigate the most important determinants of neonatal outcomes.

#### Neonatal outcomes

The following neonatal outcomes were investigated

1. Neonatal birth weight (appropriate or low birth weight): Low Birth Weight (LBW) is defined as weight at birth that is between 1500 g and 2499 g, or lower than 1500 g as per the World Health Organization (WHO)^13^.
2. Gestational age at the time of birth: Neonates were considered preterm if birth was before completing 37 weeks of gestation, or less than 259 days since the first day of last menstrual period (LMP)^14^.
3. Neonatal COVID-19 infection: Neonate with positive swab using PCR test within 48 h of delivery
4. NICU admission: Admission to the NICU within the first week of delivery.

### Statistical analysis

Data analysis was performed using the Statistical Package of Social Sciences (SPSS, version 26.0). Continuous variables were presented as mean ± standard deviation (SD), while categorical variables were described as frequency (percentage). Chi square, Fisher exact test and independent t test were used to compare between both groups of the participants as appropriate. Relative risk was used to express the impact of severity of illness on neonatal outcomes. Multivariate logistic regression was performed to determine the associations between participants’ characteristics (Age, BMI, Parity, and Severity of COVID-19 Infection) and neonatal outcomes. For logistic regression, entry was set at 0.05 and removal at 0.1 using backward Wald. Odds ratios (OR) and adjusted OR (aOR) with 95% confidence interval (CI) were also estimated. Checking for collinearity and all regression requirements was carried out before regression analysis. A p-value of < 0.05 was considered statistically significant.

## Results

### Demographic characteristics

A total of 270 patients were screened for incusion and only 200 patients fulfilled the inclusion criteria. The other 70 patients were excluded as they had comorbidities in addition to the COVID-19, which could be a confounder that leads to bias in the study. Table 1 represents patients’ demographic characteristics. Majority of the pregnant women had asymptomatic or mild infection (73.5%, n = 147), and 26.5% (n = 53) were classified as moderate to severe form of the infection. Most of the patients were from the Middle East Asian region with a mean age of 28.5 ± 1.4 and an average BMI of 26.1 ± 2.8 kg/m^2^. More than two thirds (74.0%, n = 148) of the pregnant women were multigravida. There was a significant difference between the studies groups in term of age, BMI, and parity (p < 0.001). Further details are illustrated in Table 1.

**Table 1:** Demographic characteristics of the infected pregnant women with COVID-19.

Variables	All patients (n = 200)	Asymptomatic/mild (n = 147)	Moderate/severe (n = 53)	p-value
Age (mean ± SD)	28.5 ± 1.4	27.5 ± 4.5	31.4 ± 3.3	< 0.001*
BMI (mean ± SD)	26.1 ± 2.8	25.6 ± 2.6	27.6 ± 2.3	< 0.001*
**Ethnicity, n (%)**				
Middle East Asian	121 (60.5)	89 (60.5)	32 (60.4)	0.555
Others	79 (39.5)	58 (39.5)	21 (39.6)	
**Parity, n (%)**				
Primigravida	52 (26.0)	49 (33.3)	3 (5.7)	< 0.001*
Multigravida	148 (74.0)	98 (66.7)	50 (94.3)	

*Significance at p-value < 0.05.

### Impact of severity of COVID-19 on neonatal outcomes

Table 2 discusses the obstetric and neonatal outcomes after COVID-19 infection among pregnant women. Among the whole study subjects, 35.0% (n = 70) pregnant women had preterm delivery, 30.5% (n = 61) of the neonates had low birth weight, 17.0% (n = 34) had neonatal infection, and 33.0% (n = 66) of the neonates were admitted to the ICU.

**Table 2:** Neonatal outcomes.

Groups/outcomes	Gestational age at delivery, n (%) Preterm	Neonatal birth weight, n (%) Low birth weight	Neonatal infection, n (%) Infected	Neonatal ICU admission, n (%) Admitted
All patients (n = 200)	70 (35.0)	61 (30.5)	34 (17.0)	66 (33.0)
Asymptomatic/mild (n = 147)	23/147 (15.6)	14/147 (9.5)	3/147 (2.0)	14/147 (9.5)
Moderate/severe (n = 53)	47/53 (88.7)	47/53 (88.7)	31/53 (58.5)	52/53 (95.0)
Relative risk	5.7	9.3	29.3	10
p-value**	< 0.001*	< 0.001*	0.001*	< 0.001*

*Significance at p-value < 0.05.

**Fisher Exact Test (*X*^2^).

Majority of the pregnant women in the Moderate/Severe group had shorter gestational age (preterm delivery) with p < 0.001 and 5.7 times the risk as compared to Asymptomatic/Mild group. In addition, most of the neonates among the Moderate/Severe had low birth weight with 9.3 times the risk compared to the Asymptomatic/Mild group (p < 0.001). Moreover, it was observed that the incidence rate of neonatal infection and ICU admission was much higher among the Moderate/Severe compared to Asymptomatic/Mild group (RR = 29.3, p = 0.001 and RR = 10, p < 0.001; respectively).

Logistic regression analysis was then conducted to identify the most important predictors of neonatal outcomes. The following variables were entered into logistic regression: age, BMI, Parity, Severity of COVID-19 infection. Based on the multivariate logistic regression analysis, pregnant women with moderate to severe COVID-19 were at much higher risk of preterm delivery, lower birth weight, neonatal infection as well as neonatal ICU admission (p < 0.001). In addition, multigravida women were at higher risk for preterm delivery and lower birth weight (p = 0.017 and p = 0.02; respectively) as shown in Table 3.

**Table 3:** Predictive factors of neonatal outcomes based on of logistic regression analysis.

Variables	Standard error (S.E)	Odd ratio (OR)	Confidence interval (CI 95.0%)		p-value
			Lower	Upper	
**Gestational age at delivery**					
Severity	0.50	34.49	13.04	91.19	< 0.001
Parity	0.59	4.04	1.28	12.71	0.017
**Neonatal birth weight**					
Severity	0.53	62.31	22.09	175.75	< 0.001
Parity	0.78	6.09	1.33	27.89	0.020
**Neonatal infection**					
Severity	0.66	51.67	14.32	186.45	< 0.001
**Neonatal ICU admission**					
Severity	1.05	494.00	63.35	3852.43	< 0.001

## Discussion

To best of our knowledge, this is the first study conducted in multicenter facilities in the UAE investigating the relationship between the severity of COVID-19 and obstetric/neonatal outcomes among pregnancies. Pregnant women in the 2nd trimester were included in this study and followed up till delivery. Patients were stratified into asymptomatic/mild and moderate to severe cases. Majority of the pregnant women had mild symptoms of COVID-19 (73.5%) and only 26.5% had moderate to severe manifestation. Consistently, a previous study reported 86.0% mild cases and around 14.0% between severe and critical cases among pregnant women infected with COVID-19^15^. Additionally, similar proportions were observed among the general population^16^. Pregnancies with severe form of COVID-19 were more likely to present with adverse maternal and neonatal outcomes, which were defined by preterm delivery, low neonatal birth weight, neonatal infection and/or admission to the NICU. Overall, incidence rates of the abovementioned outcomes were significantly high among patients with severe clinical manifestation compared to those with uncomplicated course of the disease. Similarly, several studies indicated an increased risk of adverse obstetric and neonatal outcomes among pregnant women with severe COVID-19^17,18^.

The present study showed 35.0% preterm births and 30.5% low birth weight among pregnancies with COVID-19 regardless the severity of the disease. Likewise, a systematic review and meta-analysis reported an estimation of 35.0% preterm delivery^19^, while a previous study conducted in Turkey reported lower percentages of these outcomes (26.4% and 12.8%; respectively)^20^. However, a systematic review of collective nine studies showed higher rates of preterm gestational delivery (63.8%) and low birth weight (42.8%) among infected pregnancies with COVID-19^21^. On the other hand, when the obstetric and neonatal outcomes were compared between moderate to severe cases of COVID-19 and asymptomatic/mild cases, a significant increase in the risk of these outcomes and high odds ratio of preterm delivery and low birth weight (88.7%) were clearly observed among pregnancies having severe manifestation. This is consistent with the evidence which declared a significant association between severe form of COVID-19 in pregnancy and iatrogenic preterm delivery (75.0%); particularly in the third trimester^22^. Likewise, a systematic review included 31,016 pregnant women from 62 studies supported our findings showing around two folds increase in preterm labor and low birth weight among pregnancies with severe COVID-19 symptoms compared to control^23^. These consequences can be related to the maternal pneumonia during the COVID-19 course, which is the main contributing factor of pregnancy complications including preterm labor, placental abruption and possible maternal or fetal death^24^. Most of reported data showed higher rates of hospital admissions among pregnant women with COVID-19 during the late pregnancy (2nd or 3rd trimester), which emphasized the importance of strict social distancing among pregnant women, particularly at the third trimester, as well as accomplishing intensive practice to avoid acquiring infections at any stage of pregnancy^25^. In addition, if the pregnant women got infected by SARS-COV-2, early detection and necessary measures should be accounted to alleviate COVID-19 pregnancy-related outcomes.

Based on our findings, SARS-COV-2 manifested more than half of the cases (58.5%) presenting with positive throat’s swab of SARS-COV-2. Consistently, Zamaniyan et al. and Alzamora et al*.* presented similar findings, which indicates the possibility of vertical maternal-fetus transmission^26,27^. It is suggested the possibility of intrauterine vertical transmission either trans-placentally through the putative surface receptor of sensitive cells for SARS-CoV-2, which is the Angiotensin-converting enzyme-2 (ACE2)^28,29^. Another possible way of SARS-CoV-2 transmission is through placental barrier damage, which is caused by severe maternal hypoxia in pregnant with COVID-19 leading to intrauterine infection^30^. This observation was also reported by several studies; however, most of the infected neonates were only mild during the prenatal period^31,32^. Furthermore, a previous case report presented a pregnant woman without unsuspected COVID-19 symptoms delivered a fetus with COVID-19 typical symptoms and confirmed positive PCR test, which demonstrates that vertical transmission can highly occur during the late pregnancy (3rd trimester) or upon delivery even if the mother have non-specific symptoms of COVID-19^33^. This case spotlights the value of regular COVID-19 screening of pregnant women for specific and nonspecific symptoms of the disease prior delivery to account all required precautions and ensure safety of mothers, newborns, as well as medical staff. On the other hand, some case reports confirmed the possibility of vertical transmission due to elevated concentration of SARS-COV-2 specific antibody (IgG or IgM) in the neonatal blood serum similar to that in the infected maternal blood despite that respiratory specimens were negative^34,35^. This can be justified by transplacental transmission of SARS-CoV-2 IgG antibodies from mother to fetus that are inactively induced by the presumed neonatal infection^31,35^.

To consider whether neonatal infection was acquired pre- or post-delivery, it is important to know that in addition to intrauterine vertical transmission, viral infection can be acquired during passage of the fetus through the birth canal, post-partum breastfeeding, skin contact, or inhalation of respiratory droplets of mothers or any surrounding person to the baby by coughing or sneezing^24^. On the other hand, one previous study investigated the placenta and cord blood within the first 12 h after delivery in one infected fetus through the viral nucleic acid test and results were negative for SARS-CoV-2, which indicated that intrauterine vertical transmission of the infection might not occur and there is limited evidence to support this claim^7^. One study conducted by Dong et al. indicated that the serological test for COVID-19 in neonates may appear negative in some cases, however, evidence of SARS-COV-2 infection may be delayed and detected up to 3–7 days of acquiring the infection^35^. These conflicting findings highlight the need of further research to examine the infants’ serological and immunological characteristics whose mothers are infected with SARS-COV-2 and additional investigations to determine the possibility of vertical transmission.

Unprecedently, the present study observed remarkably high proportion of NICU admission in 95.0% of the case with moderate to severe COVID-19. Early evidences reported lower rates of NICU ranging between 10.0 to 76.9%^21,29,36,37^. Although several studies anticipated vertical infection transmission and many neonates were admitted to the NICU, the infection was not confirmed in all admitted cases. This study has revealed a high number of NICU admissions for newborn of mothers with moderate/severe infection, this can be justified as majority of newborn of mothers with moderate or severe COVID-19 were preterm and the main purpose for NICU admission was for observation even if they were not positive for COVID-19 infection. Therefore, it was misleading whether newborns were admitted to NICU for COVID-19 management if they tested positive or just for isolation purposes if their PCR test was negative. According to the case series findings of Zhu et al., majority of the NICU admissions were due to premature birth and the need for respiratory support^38^.

The current study revealed that severity of the infection and parity were significantly associated with the adverse obstetric and neonatal outcomes of COVID-19. This study confirmed that pregnant women with moderate to severe infection were more likely to be older by approximately 3 to 4 years than those with asymptomatic or having mild symptoms of the disease^23,36^. Moreover, our study reported a significant association between parity (being multigravida) and developing adverse obstetric outcomes of COVID-19, which confirms Samadi et al*.* observations^18^. Nevertheless, results from a previous systematic analysis contradicted this finding^36^. Additionally, there was no impact of ethnicity on the obstetric outcomes. Contrary, several Asian studies presented higher estimations of preterm delivery and other COVID-19 adverse outcomes on pregnancy^19^.

### Study limitations

This study has several limitations including the retrospective design, relatively small sample size, ethnicity of the study populations which could impact the generalization of the results, as well as the fact that it cannot be assured whether newborns were admitted to NICU for COVID-19 management if they tested positive or just for isolation purposes if their PCR test was negative.

## Conclusion

The present study revealed that the severity of COVID-19 infection and multigravida have substantial influence on adverse neonatal outcomes such as preterm labor, low birth weight, neonatal infection and NICU admission. Strategies should be investigated to reduce the risk of adverse neonatal outcomes in pregnant women with COVID-19 infection.

## Abbreviations

NICU: Neonatal intensive care unit
SARS-CoV-2: Severe acute respiratory syndrome coronavirus 2
CDC: Centres of Disease Control and Prevention
COVID-19: SARS CoV-2 infection disease-19
CRP: C-Reactive Protein
DSREC: Dubai Scientific Research Ethics Committee
WHO: World Health Organization
RT PCR: Real-time polymerase chain reaction
NIH: National Institute of Health
SpO2: Oxygen saturation
LBW: Low birth weight
LMP: Last menstrual period
SPSS: Statistical package of social sciences
SD: Standard deviation
OR: Odds ratios
aOR: Adjusted OR
CI: Confidence interval
ACE2: Angiotensin-converting enzyme-2

## References

[1] Alfaraj SH, Al-Tawfiq JA, Memish ZA (2019). Middle East Respiratory Syndrome Coronavirus (MERS-CoV) infection during pregnancy: Report of two cases & review of the literature. J. Microbiol. Immunol. Infect. Wei mian yu gan ran za zhi.

[2] Vouga M (2021). Maternal outcomes and risk factors for COVID-19 severity among pregnant women. Sci. Rep..

[3] Ellington S (2020). Characteristics of women of reproductive age with laboratory-confirmed SARS-CoV-2 infection by pregnancy status—United States, January 22–June 7, 2020. MMWR Morb. Mortal. Wkly. Rep..

[4] Vousden N (2021). The incidence, characteristics and outcomes of pregnant women hospitalized with symptomatic and asymptomatic SARS-CoV-2 infection in the UK from March to September 2020: A national cohort study using the UK Obstetric Surveillance System (UKOSS). PLoS ONE.

[5] Chen H (2020). Clinical characteristics and intrauterine vertical transmission potential of COVID-19 infection in nine pregnant women: A retrospective review of medical records. Lancet.

[6] Yan J (2020). Coronavirus disease 2019 in pregnant women: A report based on 116 cases. Am. J. Obstet. Gynecol..

[7] Yu N (2020). Clinical features and obstetric and neonatal outcomes of pregnant patients with COVID-19 in Wuhan, China: A retrospective, single-centre, descriptive study. Lancet. Infect. Dis.

[8] Zhang L (2020). Analysis of the pregnancy outcomes in pregnant women with COVID-19 in Hubei Province. Zhonghua Fu Chan Ke Za Zhi.

[9] Taghavi SA (2021). Obstetric, maternal, and neonatal outcomes in COVID-19 compared to healthy pregnant women in Iran: A retrospective, case-control study. Middle East Fertil. Soc. J..

[10] Liu Y, Chen H, Tang K, Guo Y (2020). Withdrawn: Clinical manifestations and outcome of SARS-CoV-2 infection during pregnancy. J. Infect..

[11] Della Gatta AN, Rizzo R, Pilu G, Simonazzi G (2020). Coronavirus disease 2019 during pregnancy: A systematic review of reported cases. Am. J. Obstet. Gynecol..

[12] NIH. *Overview of COVID-19*, https://www.covid19treatmentguidelines.nih.gov/overview/overview-of-covid-19/ (2021). Accessed 5 Nov 2021.

[13] WHO. *ICD-11 for Mortality and Morbidity Statistics* https://icd.who.int/browse11/l-m/en#/http://id.who.int/icd/entity/2041060050 (2019). Accessed 5 Nov 2021.

[14] Howson CP, Kinney MV, McDougall L, Lawn JE, Born Too Soon Preterm Birth Action, G (2013). Born too soon: Preterm birth matters. Reprod. Health.

[15] Aghaamoo S, Ghods K, Rahmanian M (2021). Pregnant women with COVID-19: The placental involvement and consequences. J. Mol. Histol..

[16] Breslin N (2020). Coronavirus disease 2019 infection among asymptomatic and symptomatic pregnant women: Two weeks of confirmed presentations to an affiliated pair of New York City hospitals. Am. J. Obstet. Gynecol. MFM.

[17] Yang R (2020). Pregnant women with COVID-19 and risk of adverse birth outcomes and maternal-fetal vertical transmission: A population-based cohort study in Wuhan, China. BMC Med..

[18] Samadi P, Alipour Z, Ghaedrahmati M, Ahangari R (2021). The severity of COVID-19 among pregnant women and the risk of adverse maternal outcomes. Int. J. Gynaecol. Obstet..

[19] Dubey P (2021). Current trends and geographical differences in therapeutic profile and outcomes of COVID-19 among pregnant women—A systematic review and meta-analysis. BMC Pregnancy Childbirth.

[20] Oncel MY (2021). A multicenter study on epidemiological and clinical characteristics of 125 newborns born to women infected with COVID-19 by Turkish Neonatal Society. Eur. J. Pediatr..

[21] Smith V (2020). Maternal and neonatal outcomes associated with COVID-19 infection: A systematic review. PLoS ONE.

[22] Pierce-Williams RAM (2020). Clinical course of severe and critical coronavirus disease 2019 in hospitalized pregnancies: A United States cohort study. Am. J. Obstet. Gynecol. MFM..

[23] Lassi ZS (2021). A systematic review and meta-analysis of data on pregnant women with confirmed COVID-19: Clinical presentation, and pregnancy and perinatal outcomes based on COVID-19 severity. J. Glob. Health.

[24] Schwartz DA, Graham AL (2020). Potential maternal and infant outcomes from (Wuhan) coronavirus 2019-nCoV infecting pregnant women: Lessons from SARS, MERS, and other human coronavirus infections. Viruses.

[25] RCOG. *Coronavirus (COVID-19) Infection in Pregnancy*, https://www.rcog.org.uk/globalassets/documents/guidelines/2021-02-19-coronavirus-covid-19-infection-in-pregnancy-v13.pdf (2021). Accessed 8 Nov 2021.

[26] Zamaniyan M (2020). Preterm delivery, maternal death, and vertical transmission in a pregnant woman with COVID-19 infection. Prenat. Diagn..

[27] Alzamora MC (2020). Severe COVID-19 during pregnancy and possible vertical transmission. Am. J. Perinatol..

[28] Valdes G (2006). Distribution of angiotensin-(1–7) and ACE2 in human placentas of normal and pathological pregnancies. Placenta.

[29] Juan J (2020). Effect of coronavirus disease 2019 (COVID-19) on maternal, perinatal and neonatal outcome: Systematic review. Ultrasound Obstet. Gynecol..

[30] Wang C, Zhou YH, Yang HX, Poon LC (2020). Intrauterine vertical transmission of SARS-CoV-2: What we know so far. Ultrasound Obstet. Gynecol..

[31] Zeng H (2020). Antibodies in infants born to mothers with COVID-19 pneumonia. JAMA.

[32] Zeng L (2020). Neonatal early-onset infection with SARS-CoV-2 in 33 neonates born to mothers with COVID-19 in Wuhan, China. JAMA Pediatr..

[33] Naseh A, Ashrafzadeh S (2021). Possible vertical transmission from an unsuspected SARS-CoV-2-infected mother to her newborn. Cureus.

[34] Vendola N (2020). Vertical transmission of antibodies in infants born from mothers with positive serology to COVID-19 pneumonia. Eur. J. Obstet. Gynecol. Reprod. Biol..

[35] Cheng Y (2020). Kidney disease is associated with in-hospital death of patients with COVID-19. Kidney Int..

[36] Allotey J (2020). Clinical manifestations, risk factors, and maternal and perinatal outcomes of coronavirus disease 2019 in pregnancy: Living systematic review and meta-analysis. BMJ.

[37] Zaigham M, Andersson O (2020). Maternal and perinatal outcomes with COVID-19: A systematic review of 108 pregnancies. Acta Obstet. Gynecol. Scand..

[38] Zhu H (2020). Clinical analysis of 10 neonates born to mothers with 2019-nCoV pneumonia. Transl. Pediatr..

